# Validation of pore network modeling for determination of two-phase transport in fibrous porous media

**DOI:** 10.1038/s41598-020-74581-0

**Published:** 2020-11-30

**Authors:** Xiang Huang, Wei Zhou, Daxiang Deng

**Affiliations:** 1 College of Mechanical Engineering and Automation, Huaqiao University, Xiamen, 361021 China; 2 Department of Mechanical and Electrical Engineering, Xiamen University, Xiamen, 361005 China; 3 School of Mechanical Engineering and Automation, Harbin Institute of Technology, Shenzhen, 518055 China

**Keywords:** Metals and alloys, Computational methods

## Abstract

Pore network modeling (PNM) has been widely investigated in the study of multiphase transport in porous media due to its high computational efficiency. The advantage of PNM is achieved in part at the cost of using simplified geometrical elements. Therefore, the validation of pore network modeling needs further verification. A Shan-Chen (SC) multiphase lattice Boltzmann model (LBM) was used to simulate the multiphase flow and provided as the benchmark. PNM using different definitions of throat radius was performed and compared. The results showed that the capillary pressure and saturation curves agreed well when throat radius was calculated using the area-equivalent radius. The discrepancy of predicted phase occupations from different methods was compared in slice images and the reason can be attributed to the capillary pressure gradients demonstrated in LBM. Finally, the relative permeability was also predicted using PNM and provided acceptable predictions when compared with the results using single-phase LBM.

## Introduction

Porous media play a critical role in many engineering applications, such as the gas diffusion layer, micro porous layer, and other porous components^1,2^ (i.e., catalyst support layer) applied in fuel cells. The understanding of two-phase transport behavior in porous media is essential for the improvement of their performances. The mass transport in porous media depends on many factors. Besides the fluid properties (i.e., density, viscosity, capillarity, gravity), the structure of the pore space, with important characterizing parameters such as the porosity, the distribution of pore size, and the spatial connectivity of pore space also show significant impacts^3^.

Recent advances in material imaging, for example, Micro-CT, and the increase of computational power allow direct analysis of the two-phase transport using image-based methods. One of the popular analysis methods is the multiphase lattice-Boltzmann method (LBM), which is numerically solved based on the lattice and therefore coupled with 3D images naturally. Several multiphase LB models have been proposed in the past decades, such as the pseudo-potential model^4–6^, the free energy model^7^, and the color gradient model^8^. Among these models, the pseudo-potential model introduces a pseudopotential to account for particle interactions and has been widely used for its simplicity^9^. The defect of LBM, and multi-phase LBM especially, is that it can only reflect limited pore scale volume due to considerable computation expense. However, it is still invaluable in providing faithful and precise predictions of the distributions of two immiscible fluids in porous media^4^.

Pore network modeling is an attractive alternative for flow modeling, which is mostly composed of simplified spherical pore cells and tubular throats^10,11^. The flow properties can then be straight predicted from cylindrical capillaries. More realistic pore and throat representations using triangular or square^12,13^ also appear which allow for the modeling of wetting-phase trapping in corners, and the shape of pore and throat is further described in terms of a dimensionless parameter named shape factor^14^. Moreover, the effective radius which equals the radius of circle with the same cross section area was also proposed for throat shape characterization^15^. In a word, PNM is an efficient tool which is less rigorous than LBM but can provide an understanding of the relation between pore scale characteristics and engineering scale properties. However, the impacts of using different throat characterization methods on the predicted results are seldom investigated.

The accuracy of predicted results using PNM is commonly evaluated by comparison with experimental observations^12,13,15–17^. Generally, the experiments can provide valuable insight into liquid water distribution but requires advanced facilities and sophisticated skills in the design of the experiments and hence cannot be broadly accessed. On the other hand, numerical investigations, such as LBM can provide faithful results with proper parameter settings and provide as the benchmark for comparison. However, to the best of our knowledge, detailed comparisons between LBM and PNM is scarcely reported^18^.

The aim of this work is thus to evaluate the accuracy of PNM using different definitions of throat radius by benchmarking it with LBM simulated results in terms of predicted two-phase flow transport properties. We chose porous metal fibrous sintered sheet (PMFSS), a promising non-woven fibrous porous media successfully applied in fuel cells as the catalyst support layer for this study. The different approaches were evaluated with respect to their predicted relationships between saturation and capillary pressures, their phase distributions at certain saturation, and the relative permeabilities.

## Methodology

### Stochastic reconstruction of PMFSS

**Figure 1:**
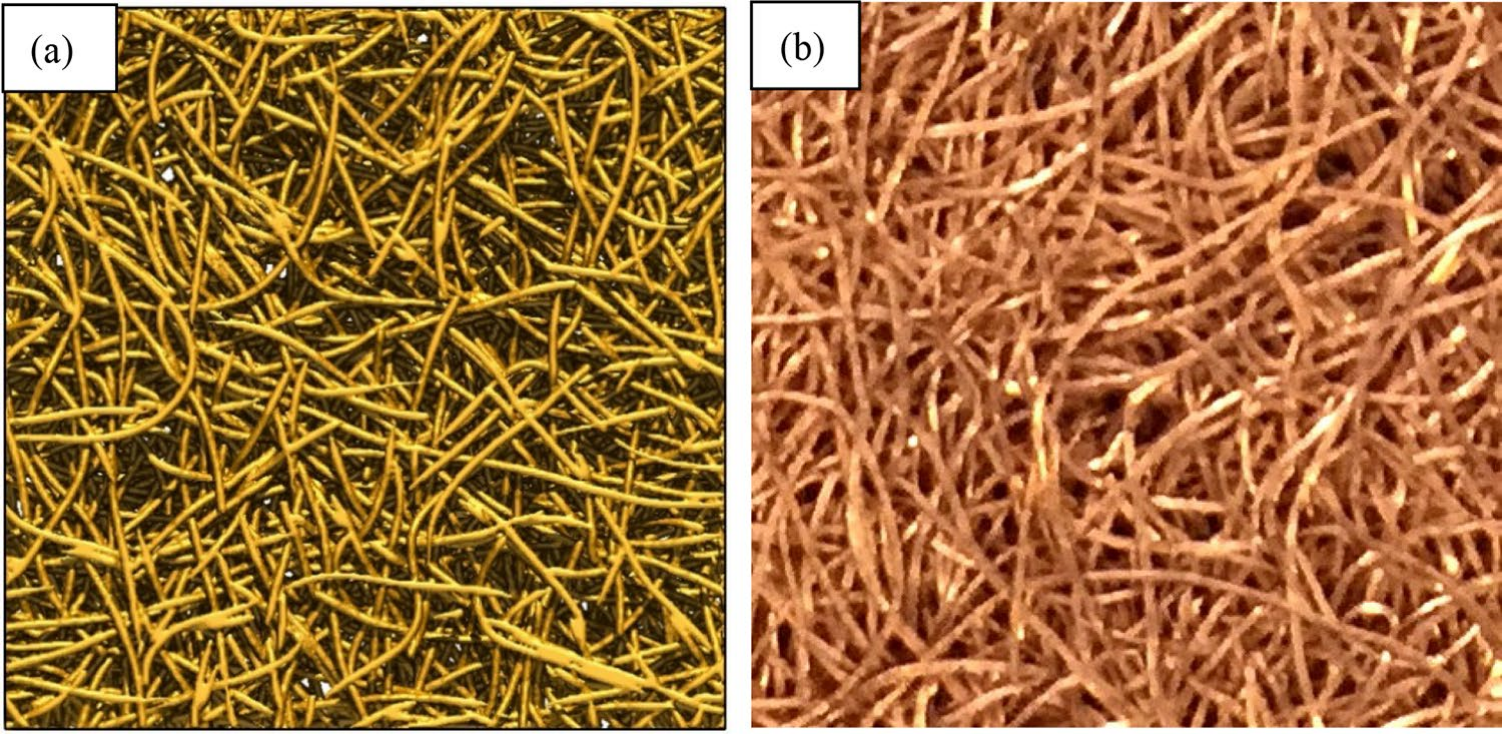
(**a**) 80% porosity stochastic PMFSS model with physical size of 8 × 8 × 2.2 mm (**b**) Optical photograph of realistic PMFSS.

A methodology for the fully customizable generation of realistic virtual nonwoven is of critical importance for its optimization design^19^. In this work, the 3D models of PMFSS are reconstructed with controllable geometric properties including porosity, fiber aspect radio (length vs. diameter), fiber orientation, curvature, pore size distributions, and degree of fiber overlapping based on morphological properties extracted from realistic PMFSS. The details of the reconstruction procedure are elaborated in our recent work^20^. Briefly, the reconstruction processes can be divided into three steps: (1) generation of curved single fiber using morphological data from 3D micro-CT images of realistic PMFSS; (2) construction of prime fiber system as accumulations of single fibers; (3) conversion from arbitrary overlapping into non-overlapping fiber system via ball-chain simulation. Figure 1 shows the reconstructed 80% porosity PMFSS model with the physical size of 8 × 8 × 2.2 mm investigated in this work. The tube-like fiber diameter is about 0.1 mm. It shows that our reconstructed stochastic model (Fig. 1a) is very similar to the photo of realistic PMFSS (Fig. 1b). To facilitate LBM and PNM, the model is constructed as 3d images with a pixel resolution of 400 × 400 × 110 (x × y × z). It’s verified that to obtain statistically valid results, the representative elementary volume (REV) required for single phase permeability simulation in PMFSS is about 1 mm in each dimension^21^. The model size in this work is much bigger and therefore should meet the statistically valid criteria.

### Shan-and-Chen (SC) pseudo-potential multi-phase model

#### Model description

We employed the SCLBM model^22^ in three dimensions for a multicomponent multiphase system. SC multicomponent LBM model is extended from the classical single component LB model by introducing distribution functions, with each one denoting the number density of one component at each lattice node. The distribution function is^23^:

1 $${\varvec{f}}_{{\varvec{i}}}^{{\varvec{k}}} \left( {{\mathbf{x}} + {\mathbf{e}}_{i} \Delta t,{\varvec{t}} + \Delta {\varvec{t}}} \right) - {\varvec{f}}_{{\varvec{i}}}^{{\varvec{k}}} \left( {{\mathbf{x}},{\varvec{t}}} \right) = \frac{{\Delta {\varvec{t}}}}{{\tau^{k} }}\left[ {f_{i}^{{k\left( {eq} \right)}} \left( {{\mathbf{x}},t} \right) - f_{i}^{k} \left( {{\mathbf{x}},t} \right)} \right],$$

where ${\varvec{f}}_{{\varvec{i}}}^{{\varvec{k}}} \left( {{\mathbf{x}},{\varvec{t}}} \right)$ is the distribution function in the *i*th velocity direction of the *k*th component, **e**_*i*_ is a lattice velocity vector, τ^*k*^ is the relaxation time of the *k*th component, which determines how quickly the system evolves to equilibrium, τ^*k*^ was empirically set to 1^24^. The right-hand of Eq. (1) represents the collision term, using the so-called BGK (Bhatnagar-Gross-Krook), or the single time relaxation approximation, the equilibrium distribution function $f_{i}^{{k\left( {eq} \right)}} \left( {{\mathbf{x}},t} \right)$ is defined as the following form (Eq. 2) for the purpose of recovering the Navier–Stokes equation for each component^25^:

2 $$f_{i}^{{k\left( {eq} \right)}} \left( {{\mathbf{x}},t} \right) = w_{i} \rho_{k} \left[ {1 + \frac{{{\mathbf{e}}_{i} \cdot u_{k}^{eq} }}{{c_{s}^{2} }} + \frac{{\left( {{\mathbf{e}}_{i} \cdot u_{k}^{eq} } \right)^{2} }}{{2c_{s}^{4} }} - \frac{{\left( {u_{k}^{eq} } \right)^{2} }}{{2c_{s}^{2} }}} \right]$$

for the D3Q19 model, the discrete velocities are given by

$${\mathbf{e}}_{i}=\left\{\begin{array}{l}\left(\mathrm{0,0},0\right), i=0\\ \left(\pm \mathrm{1,0},0\right)c,\left(0,\pm \mathrm{1,0}\right)c,\left(\mathrm{0,0},\pm 1\right)c, i=1,\dots ,6 \\ \left(\pm 1,\pm \mathrm{1,0}\right)c,\left(\pm \mathrm{1,0},\pm 1\right)c,\left(0,\pm 1,\pm 1\right)c, i=7,\dots ,18\end{array}\right.$$

$$w_{i} = \left\{ {\begin{array}{*{20}l} {\frac{1}{3}, i = 0} \\ {\frac{1}{18}, i = 1,2, \ldots ,6} \\ {\frac{1}{36}, i = 7,8, \ldots ,18,} \\ \end{array} } \right.$$

where *c*_*s*_ = *c*/$\sqrt 3$, *c* = Δ*x*/Δ*t* is the ratio of lattice spacing Δ*x* and time step Δ*t*. Both of Δ*x* and Δ*t* equals 1 in this work. *ρ*_*k*_ is the density of *i*th component, and can be obtained as: *ρ*_*k*_ = $\sum\nolimits_{i} {f_{i}^{k} }$.

*u*_*k*_^*eq*^ is the equilibrium velocity which is :

3 $$u_{k}^{eq} = u^{\prime} + \frac{{\tau_{k} {\mathbf{F}}_{k} }}{{\rho_{k} }},$$

where *u*′ is the common velocity which is calculated as

4 $$u^{\prime} = \frac{{\mathop \sum \nolimits_{k} \left( {\mathop \sum \nolimits_{i} \frac{{f_{i}^{k} {\mathbf{e}}_{i} }}{{\tau^{k} }}} \right)}}{{\mathop \sum \nolimits_{k} \frac{{\rho_{k} }}{{\tau^{k} }}}}.$$

In Eq. (3), **F**_*k*_ = **F**_*c*,*k* +_ **F**_*ads*,*k*_ is the total interaction force on the *k*th component, including fluid–fluid cohesion **F**_*c*,*k*_ and fluid–solid adhesion **F**_*ads*,*k*_

5 $${\mathbf{F}}_{c,k} \left( {{\mathbf{x}},t} \right) = - G_{c} \rho_{k} \left( {{\mathbf{x}},t} \right)\mathop \sum \limits_{i} w_{i} \rho_{{\overline{k}}} \left( {{\mathbf{x}} + {\mathbf{e}}_{i} \Delta t,t} \right){\mathbf{e}}_{i} ,$$

where *G*_c_ is a parameter that controls the strength of the cohesive force between components *k* and $\overline{k}$.

6 $${\mathbf{F}}_{ads,k} \left( {{\mathbf{x}},t} \right) = - G_{ads,k} \rho_{k} \left( {{\mathbf{x}},t} \right)\mathop \sum \limits_{i} w_{i} s\left( {{\mathbf{x}} + {\mathbf{e}}_{i} \Delta t,t} \right){\mathbf{e}}_{i} .$$

Similar to the fluid cohesion parameter, *G*_*ads*,*k*_ controls the strength of the interaction between component *k* and the solid. *s* is an indicator function which equals to 1 for a solid node or 0 for a fluid node.

#### Parameter determination and model validation

Before investigating the physical property using the SC model, the non-dimensional parameters, involving fluid–fluid and fluid–solid interaction coefficients should be calibrated. For this purpose, two numerical experiments, named bubble test and flow through capillary tube were conducted.

##### Bubble test

Test was conducted for 3D bubbles. The domain size was 51 × 51 × 51 lattice units with initial spherical bubbles of various radius. The initial density value of fluid-1 (*ρ*_1_) equals 8 inside the bubble and 0.014 outside the bubble, while the initial density value of fluid-2 (*ρ*_2_) equals 0.014 inside the bubble and 8 outside the bubble. Fluid-1 refers to the non-wetting phase (NWP) and fluid-2 refers to the wetting phase (WP) respectively and this convention is used in the rest of this work. Periodic boundary conditions are applied in all the six borders. In the SC model, the miscible/immiscible behavior of two components depends on the cohesion strength and density value. We set *G*_c_ = 0.25, which shows good component separation with sharp interface between the two components and numerical stability. This value is in ranges of other Refs.^23,24^, where *G*_c_(*ρ*_1_ + *ρ*_2_) was close to 2.0. The main defect of SC LBM is that the interface between two fluid is several lattice units in thickness. Hence, we considered a lattice node is occupied by one fluid if its density is larger than half of its maximal value^25^.

The simulation results were evaluated against Laplace’s law (Eq. 7), which indicates that the surface tension *γ* is determined by the NWP bubble of radius *R* and pressure difference *p* between the interface.

7 $$\Delta p = \frac{2\gamma }{R}.$$

The pressure *p* can be determined from densities as^6^

8 $$p\left( {\mathbf{x}} \right) = \left[ {\rho_{{1}} \left( {\mathbf{x}} \right) \, + \rho_{{2}} \left( {\mathbf{x}} \right)} \right]/{3} + G_{{\text{c}}} \rho_{{1}} \left( {\mathbf{x}} \right)\rho_{{2}} \left( {\mathbf{x}} \right)/{6}{\text{.}}$$

**Figure 2:**
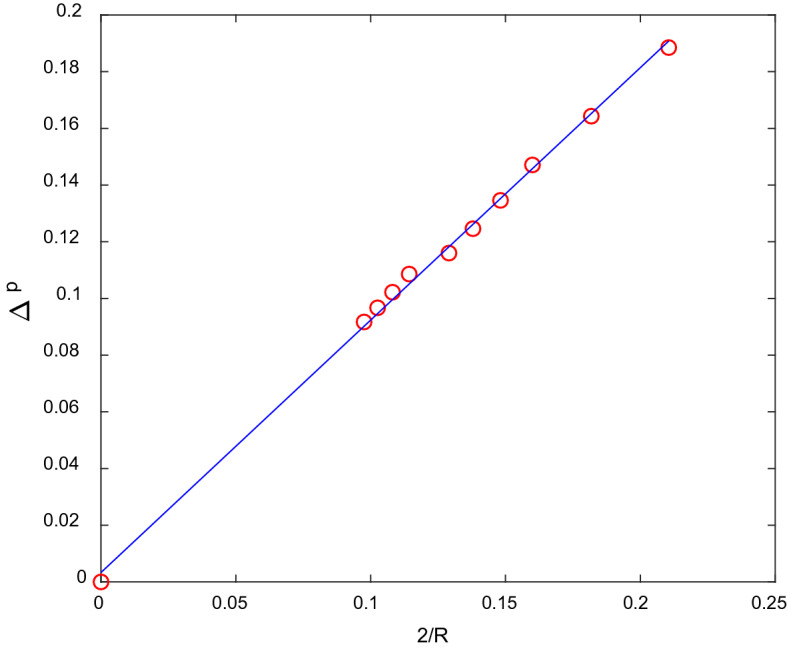
Test of Laplace’s law.

Figure 2 plots the relationship between pressure difference Δ*p* and reciprocal of *R*. It shows that Δ*p* is inversely proportional to the reciprocal of the bubble radius, which agrees well with Laplace’s law. The linear fitting result is 0.89, which is the lattice surface tension.

##### Flow through capillary tube

To investigate primary drainage process of NWP in the initially WP filled 3D tube, a 3D channel with circular cross-section of the radius of 8 lattices and length of 20 lattices is generated, with 3 additional layers of lattices of NWP and WP reservoirs at the inlet and outlet, respectively. Pressure boundary conditions are applied between inlet and outlet, while bounce back boundary condition is applied for cylindrical walls. The invasion process was then conducted by incrementally increase the inlet pressure while keeping the pressure in outlet unchanged. According to Ref.^23^, the contact angle *θ* can be evaluated as:

9 $$\cos \theta = \frac{{G_{ads,2} - G_{ads,1} }}{{G_{c} \frac{{\rho_{1} - \rho_{2} }}{2}}}.$$

**Figure 3:**
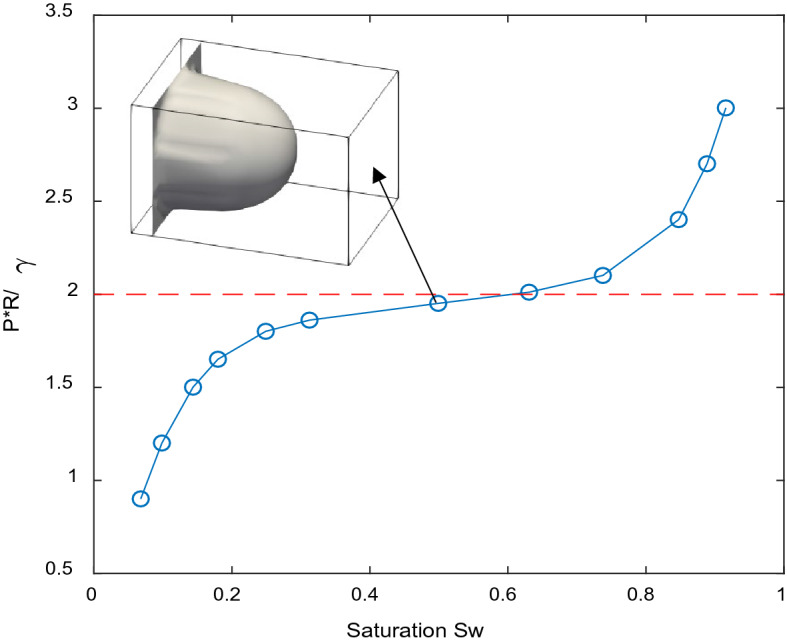
Primary drainage curves obtained by simulation of NWP displacement in a capillary tube with circular cross section.

Therefore, to ensure the invasion NWP is extremely hydrophobic (0 contact angle), as identical to the condition assumed in pore network modeling, in this work, we set *G*_*ads,2*_ = − *G*_*ads,1*_ = 0.5 according to Eq. (9). Figure 3 shows the simulated capillary pressure versus the NWP saturation curve for the capillary tube with a zero contact angle. The analytical entry capillary pressure of circular cross section tube can be obtained using Laplace’s law (Eq. 7). Good agreement was obtained between the simulated and analytical NWP entry pressure, which is the minimal pressure to drive the flow in the tube channel. The insert of Fig. 3 shows the shape and location of NWP and WP interface at 49% NWP saturation. The spherical convex curvature of the interface indicates a strong hydrophobic property.

#### Conversion from lattice unit to physical unit

The transformation into the physical unit is a key step for comparison. The physical capillary pressure can be converted from the lattice unit using the following equation^24^:

10 $$P_{c,p} = \frac{{\gamma_{p} P_{c,L} }}{{\gamma_{L} h_{p} }},$$

where *P*_*c,p*_ and *P*_*c,L*_ are the physical and lattice capillary pressure, respectively. *γ*_*p*_ and *γ*_*L*_ are the physical and lattice surface tension, respectively. *h*_*p*_ is the resolution of the lattice. All these required parameters can be accessed directly or through bobble test.

#### Implementation of the LB pressure–saturation in stochastic PMFSS

After validation of parameters, the simulation of two-phase LBM can be performed. Before that, the available computing resources should be considered. Since two-phase LBM is high time expensive, the 3D image size was reduced by doubling the node size. For 3D image, that means the number of nodes is reduced by a factor of 8 (2 × 2 × 2). This leads to the reduced 3D image of 200 × 200 × 55 pixel resolution. For NWP penetration simulation, a layer of solid wall in the 4 lateral sides parallel to the flow direction (z-axis), and buffers as 3 layers of filled NWP and WP at the inlet and outlet boundaries, respectively, were added additionally to the simulation domain. Eventually, the volume size of the simulation domain reaches 202 × 202 × 61, and the resolution of the lattice node (pixel) is 40 μm.

To obtain a pressure–saturation relationship, a group of pressure gradients was imposed between the top and bottom boundaries. The nodes at the interface of solid and void are applied with bounce back boundary conditions. Eight single step primary drainage points were performed using pressure boundary conditions. More specifically, at the inlet boundary, the 3 layers of filled NWP were initialed using NWP with increasing lattice densities of 8.25, 8.5, 8.75, 9.0, 9.125, 9.25, 9.5, and 9.75 and WP with a density of 0.014. The rest of the simulation domain, except the solid phase (fibers and 4 lateral walls), is set using densities of 0.014 and 8 for NWP and WP, respectively. This parameter sets result in physical pressure gradients of 173, 347, 520, 694, 781, 867, 1041, and 1215 Pa according to Eqs. (8) and (10). The saturation data can be evaluated as the ratio of node numbers between NWP and that of total void phase.

**Figure 4:**
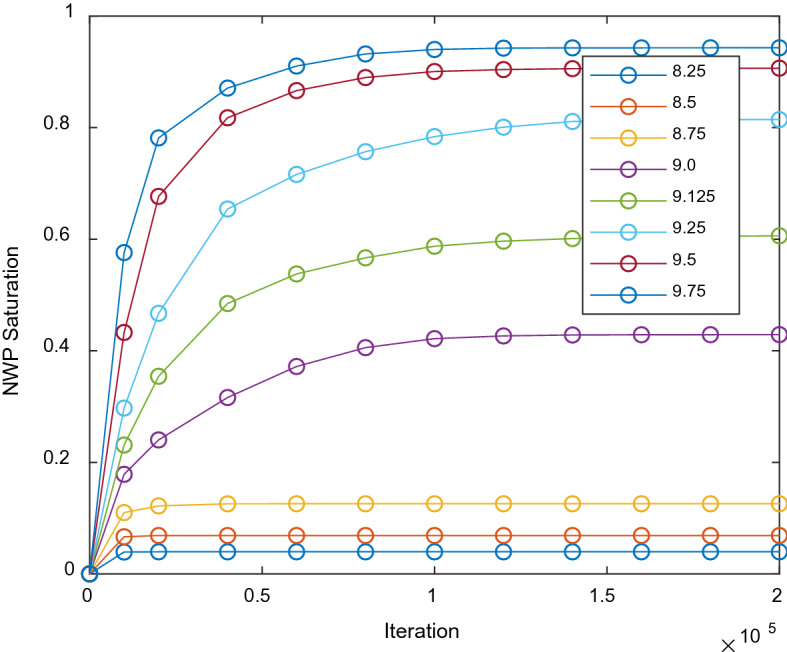
Evolution of NWP saturation in iterations of SCLBM.

To evaluate the required iterations of simulations to reach steady state, all simulations were performed with data (density and velocity of each component) recorded at fixed step intervals. As shown in Fig. 4, we detected that for high saturation the equilibrium reaches slower. Despite this issue, the saturation increases slowly after 1.5e5 iterations. Therefore, although there is no strict criterion to determine equilibrium, the saturation results should be acceptable at 2e5 iteration.

### PNM

**Figure 5:**
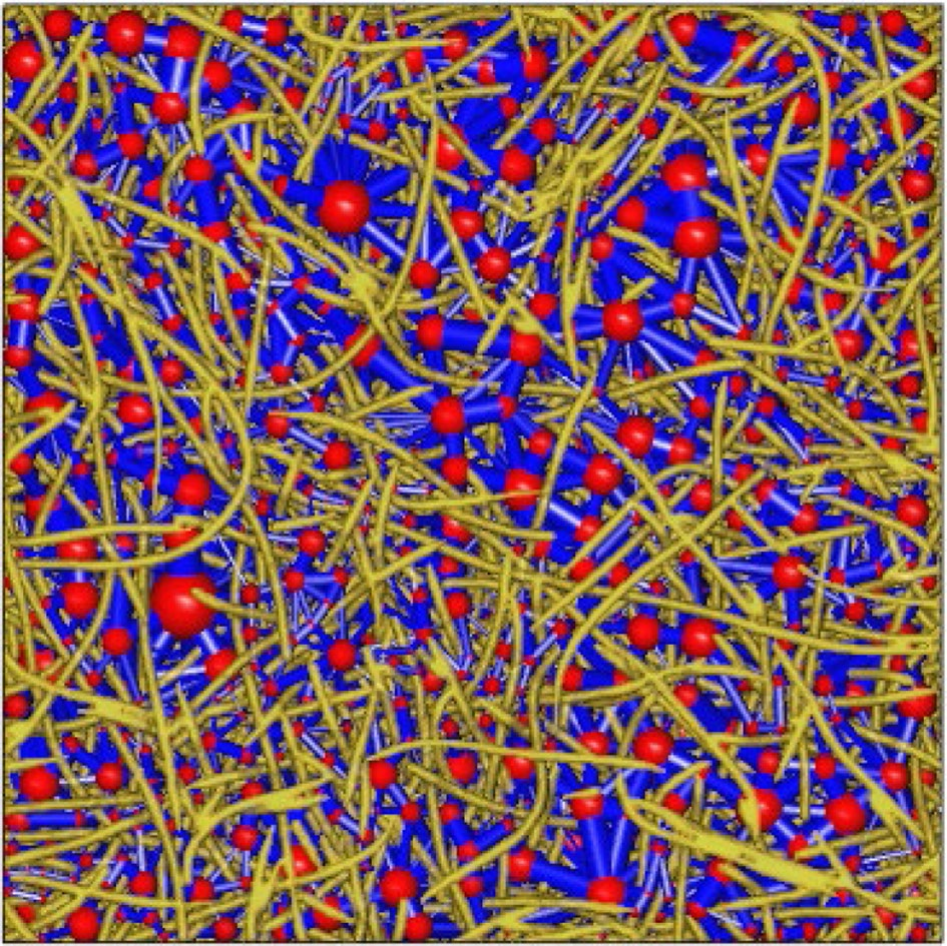
PNM of 80% porosity stochastic PMFSS model.

We use a maximal-ball labeled watershed method to extract PNM from the reconstructed 3D virtual nonwoven images (400 × 400 × 110 pixels). Figure 5 shows the extracted pore network from 3D virtual image identical to the one shown in Fig. 1. It shows that the pore space is divided into pore cells (red balls) connected by narrower throats (blue cylinders). The two-phase transport properties, involving water–air interface propagation driven by capillary pressure and relative permeability at various NWP saturations are then performed according to the invasion percolation theory. Details about pore network extraction and simulation are described in our recent work^26^. It’s notable that the lateral of the model is sealed as solid wall boundary in LBM. Therefore, to ensure the identical boundary settings, the distance values of pixels on the six boundaries except solid elements are initialed as a half pixel value.

#### Characterization of throat radius

##### Effective radius considering impact of shape factor

The primary drainage process is driven by capillary pressure. NWP invades the WP filled pore space gradually by occupying pore cells one after another, choosing the pore cell connected to the NWP filled regions connected with the throat of the lowest capillary pressures. According to Laplace’s law (Eq. 7). The invasion critical capillary pressure is determined by throat radius. Only a very small fraction of network elements will have a circular cross-section, where the throat radius can be accurately evaluated using radius of the inscribed maximal ball. For throat with an irregular shape, the capillary entry pressure can be evaluated using the Mayer & Stowe and Princen (MS-P) method, the details of the calculations are given in Refs.^12,27^:

11 $$\Delta p = \frac{{\gamma \cos \theta \left( {1 + 2\sqrt {\pi G} } \right)}}{R}F_{d} \left( {\theta ,G} \right),$$

where the definitions of parameters *p*, and *γ* are as same as in Eq. (7). *R* is the radius of the inscribed circle, which can be evaluated using the maximal distance value in the throat cross section. *θ* is the contact angle, which equals 0 in our case. *F*_*d*_ is a function depended on *θ* and *G*. According to Ref.^27^, *F*_*d*_ = 1 when *θ* = 0°. *G* is the shape factor, which is a dimensionless term to describe the irregularity of geometrical shape. Parameter *G* is usually defined as:

12 $$G = \frac{A}{{S^{2} }},$$

where *A* is the area of cross section, *S* is the corresponding perimeter length. *A* could be evaluated using pixel numbers in the throat cross section. However, perimeter *S* is not easily obtained because of the difficulty in identification of throat boundary pixels. Therefore, instead of Eq. (12), we use another evaluation proposed in Ref.^28^:

13 $$G = \frac{{R^{2} }}{4A}.$$

Finally, via comparison of Eqs. (7) and (11), we can define an effective throat radius *R*_*sf*_ of Eq. (7) considering the effect of shape factor *G*:

14 $$R_{sf} = \frac{2R}{{1 + 2\sqrt {\pi G} }}.$$

##### Area-equivalent radius

The deviation of capillary pressure evaluation using Eq. (7) directly in realistic geometry is also remedied by using an alternative means of calculation of the throat size, named equivalent diameter^15^, which equals the diameter of a circle with the same area *A* of the throat cross section, the equivalent radius accordingly is:

15 $$R_{area} = \sqrt {\frac{A}{\pi }} .$$

**Figure 6:**
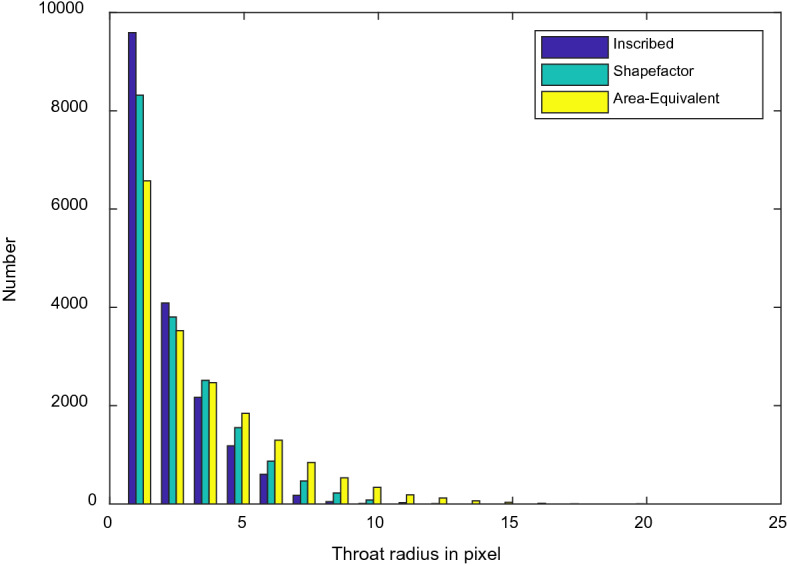
Distributions of throat radius evaluated using inscribed circle, effective radius impacted by shape factor, and area-equivalent radius.

Figure 6 shows three distributions of throat radius using inscribed maximal ball, effective radius impacted by shape factor, and area-equivalent radius. It reflects that the distributions shift right one by one from inscribed radius, shape factor radius to area-equivalent radius. This impact is also reflected in the capillary pressure and saturation relationship.

## Results

### Pressure–saturation relationship

**Figure 7:**
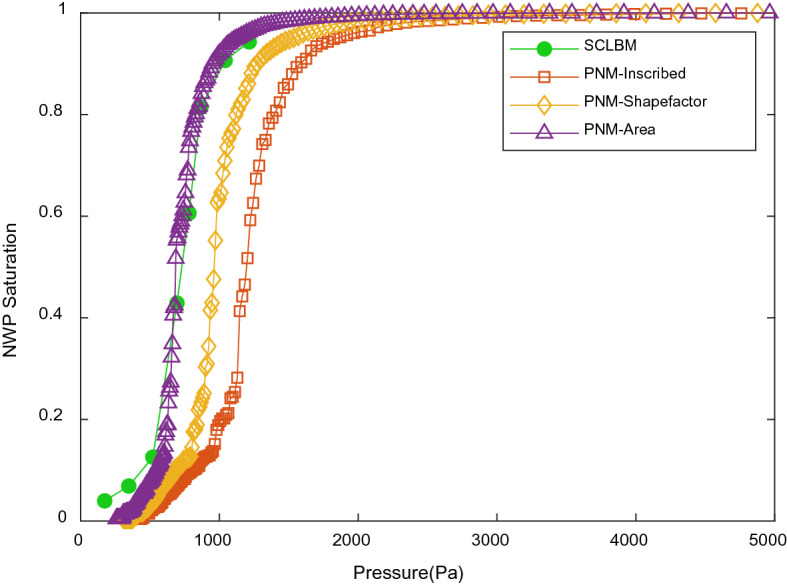
Primary drainage curves obtained by SCLBM, PNM with throat radius measured using inscribed maximal balls, effective radius impacted by shape factor and area-equivalent radius.

Capillary pressure–saturation curves are commonly used to characterize two-phase transport in porous media. The SCLBM pressure–saturation profiles along with results from PNM using different definitions of throat radius appear in Fig. 7. All simulations are performed with 0 contact angle assumptions. It can be seen from the comparison that the predicted curves are similar to each other in magnitude, shape and trend. The increase of throat radius as demonstrated in Fig. 6 results in the decrease of the breakthrough capillary pressure to obtain a given saturation, which leads to the left shift of curves with little change in the shape or slope of the curves. The curve predicted by area-equivalent radius shows excellent agreement with the SCLBM data. The good agreement between using area-equivalent radius and experimental results were also observed for similar fibrous media^15^.

Although experimental observation of PMFSS is not currently available. An analogy with the experimental measurement of a commonly explored fibrous porous media, gas diffuse layer (GDL), as reported in Ref.^16^, can still be made. The GDL material in Ref.^16^ is of similar porosity (79%), hydrophobic after teflon treatment, but of different length scales (fiber diameter of GDL is about 7 μm rather than 100 μm of PMFSS). Nevertheless, the impact caused by scale difference can be remedied considering the Young–Laplace law (Eq. 7), where the pressure is assumed to be inversely proportional to the radius of two-phase interface. Therefore, the experimental pressure value of PMFSS at certain saturation can be approximately estimated by reducing the pressure value in Ref.^16^ by a factor of 14(100/7 = 14). Specifically, the capillary pressure is about 7000 Pa at 10% water (as NWP) saturation in Fig. 12 in Ref.^16^. Accordingly, the capillary pressure is about 500 Pa at 10% NWP saturation of PMFSS, which can be detected to be in good agreement with our numerical predictions as shown in Fig. 7.

Noted that the above mentioned three types of throat radius are proposed just considering the geometrical shape of the throat cross section, despite the simplicity and accuracy of using area-equivalent radius for predicting the pressure–saturation relationship, its physical validity needs further verification. Instead, the so called CFD radius, which is defined as the radius of a tube of circular cross section that has the same resistance to the parallel flow through the throat cross section, is more physically reliable and now drawing attention^29^. However, it’s trickier as it requires one to perform a numerical solution of parallel flow through the throat cross section, and therefore is not explored in our current work.

### Comparison of NWP distributions of SCLBM and PNM

**Figure 8:**
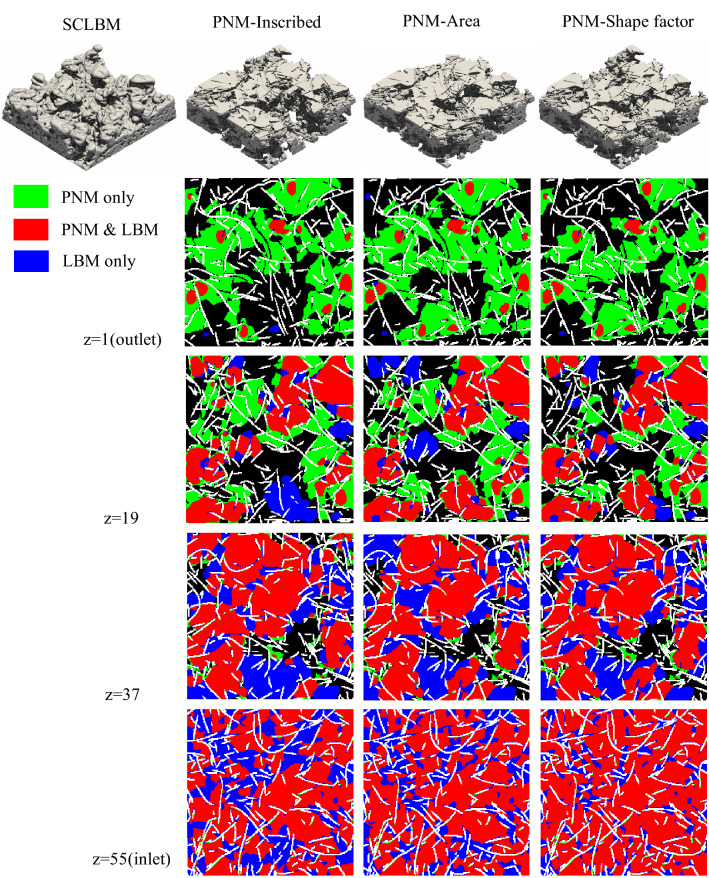
NWP distribution at 60% saturation of 3D PMFSS. The top row shows the 3D reconstruction of NWP distribution using four different methods. The slice images at different heights (z = 1, 19, 37, 55) in the invasion direction is demonstrated in the below, using different colors to distinguish components (WP: black, fiber: white, NWP predicted by PNM only: green, by PNM and LBM: red, and by LBM only: blue).

**Figure 9:**
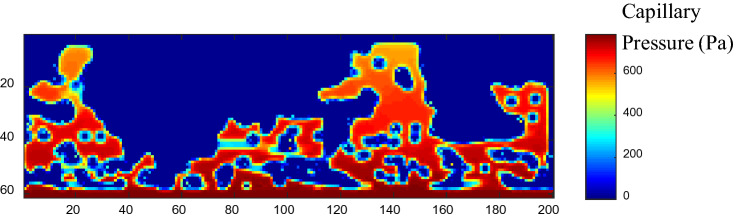
Pressure gradient generated using SCLBM at 60% NWP saturation.

The advantage of computational simulation in comparison to experimental testing is the facility of observing the local phase distributions. The NWP distributions simulated by SCLBM and PNM are compared. The top row of Fig. 8 shows the reconstructed NWP distributions at 60% NWP saturation using SCLBM, PNMs with throat characterized using inscribed circles, area-equivalent radius, and shaped factor impacted radius, respectively. The 3D reconstructed geometries of NWP from the simulation of PNMs demonstrated acceptable matches with SCLBM result. The degree of agreement between PNM and SCLBM is further presented by node occupation in chosen slices, with the red nodes denoting the matched predictions of PNM and SCLBM, the green nodes denoting PNM only predictions, and the blue nodes denoting SCLBM only results. It can be seen from the comparison that the overall agreement is acceptable with matched nodes showing the major occupation, and the locations of NWP occupied nodes predicted by different methods are close. The major deviation appears near the outlet where more PNM only occupation occurs, on the contrary, more SCLBM only occupation appears at slices closer to the inlet. This deviation can be explained in terms of the pressure field. Despite pressure field with gradient distributions can be predicted by both methods, in PNM, at the pore scale, individual pore cell is assigned with one pressure value, all throats connected to the pore cell will have equal opportunity of being invaded if their entry capillary values are close. However, the gradient pressure property is identical in all scales in the SCLBM simulation, it will lead to the priority of invading of nearby regions closer to the inlet where capillary pressure values are higher and result in the saturation gradient along the flow direction using SCLBM^30,31^. To further investigate the gradient pressure in the SCLBM, a cross section image of the capillary pressure field in range from 0 to 800 Pa of NWP at 60% saturation is shown in Fig. 9. It’s notable that the pressure values are calculated from density values (Eq. 8), therefore 2–3 layers of pressure values close to the two-phase interface are incorrect. Despite these artificial data, the gradient distribution of capillary pressure can still be obviously detected along the percolation direction. On the other hand, unlike the assumption of instant occupation of an invaded pore cell in PNM, physically, the invasion of pore element occurs gradually as reflected in LBM. Thus, a convex interface will generate at a pore element towards the percolation direction before reaching its entry capillary pressure. This will also contribute to the SCLBM only occupation near the inlet boundary.

### Relative permeability

**Figure 10:**
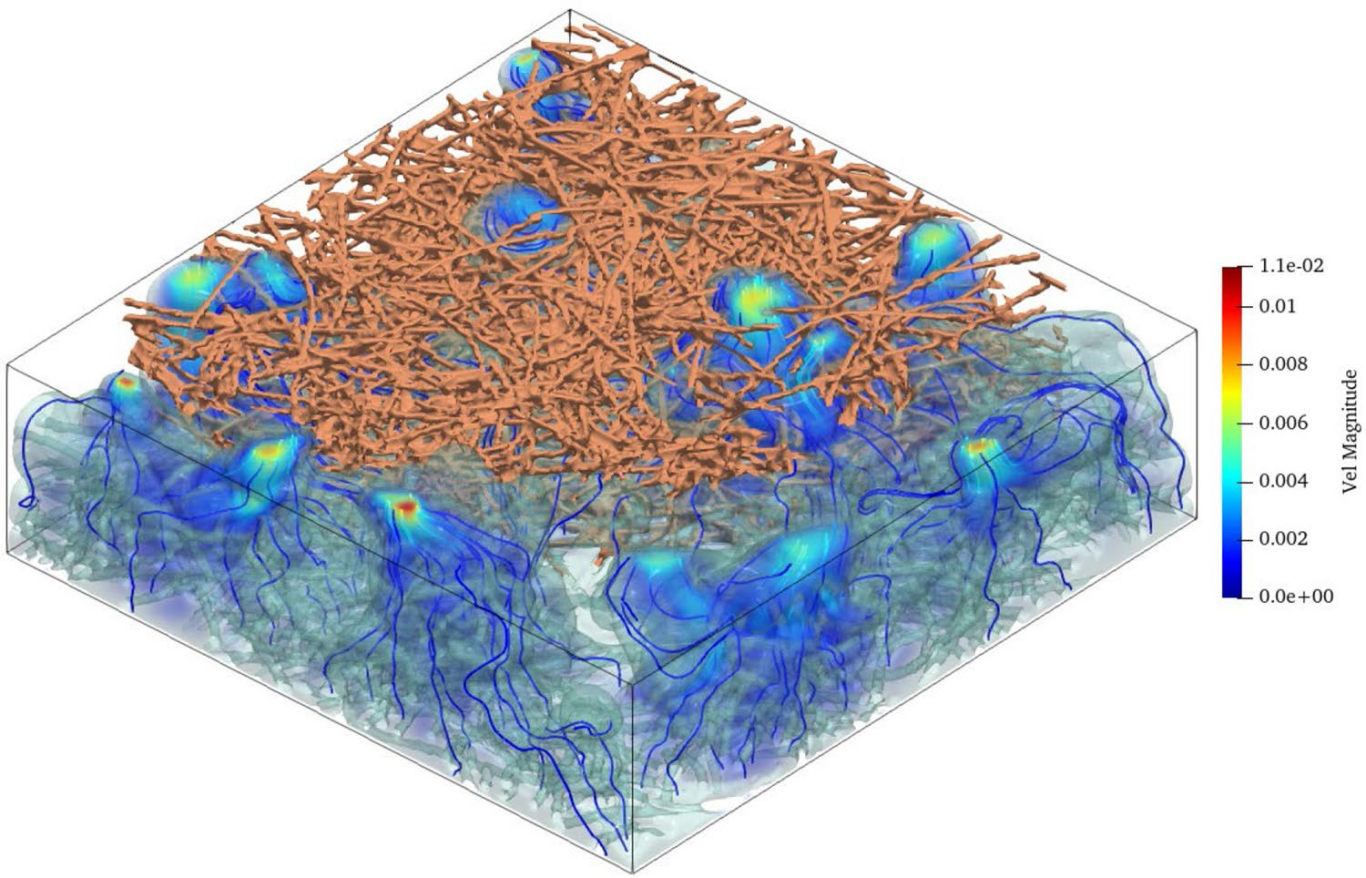
3D velocity field of NWP at 60% NWP saturation. For clarity, only a part of the solid fiber is shown. The streamlines with the color map of the flow rate magnitude indicate a significantly tortuous flow pathway of the NWP occupied pores. The highest flow rate shows at the top (outlet) surface where the bottleneck of the flow pathway is located.

**Figure 11:**
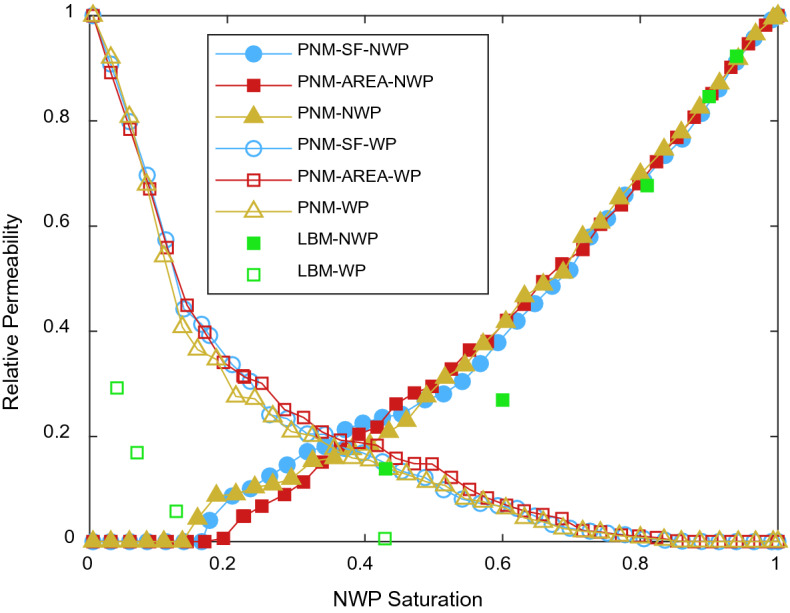
Comparison of relative permeability predicted by PNM and LBM.

The relative permeability was not accessed directly from SCLBM since the spurious velocity near the interface and thus leads to the inaccuracy of flux evaluation at the inlet and outlet boundaries^31^. Thus, we use a single-phase LBM described in Ref.^16^ to predict the relative permeabilities. Specifically, the absolute permeability is obtained through single-phase LBM simulation. Then, to access the relative permeability of NWP at certain saturation levels, the NWP distribution is firstly obtained through SCLBM. After that, the nodes occupied by NWP is considered as flow channel, and the single-phase LBM is performed in the channel. The relative permeability of the NWP can be obtained by calculating the ratio between this permeability and the absolute one. The WP permeability can be accessed similarly by treating NWP occupied nodes as solid additionally while only the WP occupied nodes forming the flow channel. Figure 10 shows the 3D velocity field of NWP at 60% NWP saturation using LBM. The streamlines with the color map of the flow rate magnitude indicate a significantly tortuous flow pathway of the NWP occupied pores. The highest flow rate occurs at the top (outlet) surface where the bottleneck of the flow pathway is located. Noted that the flow rate only exists when the saturation of the relative phase is higher than the breakthrough saturation (about 20% as shown in Fig. 11 in our case) when the flow path connecting the inlet and outlet occurs.

A comparison of the PNW relative permeability and the results obtained with LBM is presented in Fig. 11. The curves of relative permeability are close for PNM using different definitions of throat radius. The NWP relative permeability shows good agreement between the PNM and LBM, while the LBM predicts lower WP relative permeability. This deviation can be explained by the two-phase distribution as can be seen in Fig. 8, where the connected path for WP to pass from inlet to outlet is restricted due to the higher ratio of NWP occupation nodes in SCLBM results and leads to the narrower WP path compared with the PNM results.

## Conclusion

In this work the two-phase transport properties, involving capillary pressure and relative permeability in a porous fibrous media, PMFSS, are performed using SCLBM and PNMs with different definitions of throat radius. Numerical calibrations of bobble test and flow in circular cross section capillary tube were performed in advance to obtain lattice surface tension and desired contact angle with proper LBM parameters. The lattice unit was transformed into the physical unit by relating lattice and physical surface tension. The results of SCLBM are provided as benchmark data for comparison. A pore network model generated using the watershed method as discussed in our recent work was applied and the invasion percolation process was conducted based on it. The predicted capillary pressure curves show good agreement in magnitude and trend using different methods. The excellent match was observed when PNM using the area-equivalent radius was performed. The NWP distribution in slices was analyzed, and show a good overall agreement. The discrepancies between the PNM and SCLBM results were discussed, which can be partly attributed to the pressure gradient in SCLBM. Finally, the relative permeability data derived using PNM and SCLBM was compared and show good agreement of the NWP relative permeability. The disagreement in WP relative permeability is explained and in line with the observations in the discrepancies of NWP distributions near the inlet boundary between methods.

## References

[1] Peiyun Y, Linfa P, Xinmin L, Mutain L, Jun N (2012). Investigation of sintered stainless steel fiber felt as gas diffusion layer in proton exchange membrane fuel cells. Int. J. Hydrog. Energy.

[2] Wei Z (2009). A performance study of methanol steam reforming microreactor with porous copper fiber sintered felt as catalyst support for fuel cells. Int. J. Hydrog. Energy.

[3] Aurelien GM (2019). Measuring simplified pore-throat angularity using automated mathematical morphology. SPE J..

[4] Sukop MC (2008). Distribution of multiphase fluids in porous media: Comparison between lattice Boltzmann modeling and micro-X-ray tomography. Phys. Rev. E.

[5] Mukherjee PP, Wang CY, Kang QJ (2009). Mesoscopic modeling of two-phase behavior and flooding phenomena in polymer electrolyte fuel cells. Electrochim. Acta..

[6] Warda HA, Haddara SH, Wahba EM, Sedahmed M (2017). Lattice Boltzmann simulations of the capillary pressure bump phenomenon in heterogeneous porous media. J. Pet. Sci. Eng..

[7] Hao L, Cheng P (2010). Lattice Boltzmann simulations of water transport in gas diffusion layer of a polymer electrolyte membrane fuel cell. J. Power Sources.

[8] Huang JW, Xiao F, Yin XL (2017). Lattice Boltzmann simulation of pressure-driven two-phase flows in capillary tube and porous medium. Comput. Fluids.

[9] Chen L, Kang QJ, Mu YT, He YL, Tao WQ (2014). A critical review of the pseudopotential multiphase lattice Boltzmann model: Methods and applications. Int. J. Heat Mass Transf..

[10] Gostick JT, Aghighi M, Hinebaugh J (2016). OpenPNM: A pore network modeling package. Comput. Sci. Eng..

[11] Pradeep B, Clintion SW, Karsten ET (2011). Effect of network structure on characterization and flow modeling using X-ray micro-tomography images of granular and fibrous porous media. Transp. Porous Med..

[12] Valvatne PH, Blunt MJ (2004). Predictive pore-scale modeling of two-phase flow in mixed wet media. Water Res..

[13] Patzek TW (2001). Verification a complete pore network simulator of drainage and imbibition. SPE J..

[14] Patzek TW, Silin DB (2001). Shape factor and hydraulic conductance in noncircular capillaries I. One-phase creeping flow. J. Colloid Interface Sci..

[15] Gostick JT (2017). Versatile and efficient pore network extraction method using marker-based watershed segmentation. Phys. Rev. E.

[16] Koido T, Furusawa T, Moriyama K (2008). An approach to modeling two-phase transport in the gas diffusion layer of a proton exchange membrane fuel cell. J. Power Sources.

[17] Agaesse T, Lamibrac A, Buchi FN, Pauchet J, Prat M (2016). Validation of pore network simulations of ex-situ water distributions in a gas diffusion layer of proton exchange membrane fuel cells with X-ray tomographic images. J. Power Sources.

[18] Vogel HJ, Tolke J, Schulz VP, Krafczyk M, Roth K (2005). Comparison of a Lattice-Boltzmann model, a full-morphology model, and a pore network model for determining capillary pressure–saturation relationships. Vadose Zone J..

[19] Abishek S (2017). Generation and validation of virtual nonwoven, foam and knitted filter (separator/coalescer) geometries for CFD simulations. Sep. Purif. Technol..

[20] Xiang H (2017). 3D stochastic modeling, simulation and analysis of effective thermal conductivity in fibrous media. Powder Technol..

[21] Xiang H (2015). Morphology and transport properties of fibrous porous media. Powder Technol..

[22] Shan X, Chen H (1994). Lattice Boltzmann model for simulating flows with multiple phases and components. Phys. Rev. E.

[23] Haibo H, Thorne DT, Schaap MG, Sukop MC (2007). Proposed approximation for contact angles in Shan-and-Chen-type multicomponent multiphase lattice Boltzmann models. Phys. Rev. E.

[24] Schaap MG, Porter ML, Christensen BSB, Widenschild D (2007). Comparison of pressure–saturation characteristics derived from computed tomography and lattice Boltzmann simulations. Water Res..

[25] Pan C, Hilpert M, Miller CT (2004). Lattice-Boltzmann simulation of two-phase flow in porous media. Water Res..

[26] Xiang H, Yinwu H, Wei Z, Deng DX, Zhao YW (2019). Pore network modeling of fibrous porous media of uniform and gradient porosity. Powder Technol..

[27] Oren PE, Bakke S, Amtzen OJ (1998). Extending predictive capabilities to network models. SPE J..

[28] Bultreys T (2018). Validation of model predictions of pore-scale fluid distribution during two-phase flow. Phys. Rev. E.

[29] Rong LW, Dong KJ, Yu AB (2020). Lattice-Boltzmann computation of hydraulic pore-to-pore conductance in packed beds of uniform spheres. Chem. Eng. Sci..

[30] Li H, Pan C, Miller CT (2005). Pore-scale investigation of viscous coupling effects for two-phase flow in porous media. Phys. Rev. E.

[31] Huang HB, Li ZT, Liu SASA, Lu XY (2009). Shan-and-Chen-type multiphase lattice Boltzmann study of viscous coupling effects for two-phase flow in porous media. Int. J. Numer. Methods Fluid.

